# Effect of nasal irrigation in adults infected with Omicron variant of COVID-19: A quasi-experimental study

**DOI:** 10.3389/fpubh.2022.1046112

**Published:** 2023-01-09

**Authors:** Li Liu, Shuangshuang Xie, Cheng Li, Liang Su, Chengbao Zhu

**Affiliations:** ^1^Department of Digestive Diseases, Shandong Public Health Clinical Center, Shandong University, Jinan, China; ^2^Department of Medical Service, Shandong Public Health Clinical Center, Shandong University, Jinan, China; ^3^Department of Clinical Laboratory, Shandong Public Health Clinical Center, Shandong University, Jinan, China

**Keywords:** COVID-19, Omicron, nasal irrigation, clinical study, quasi-experimental study

## Abstract

**Objective:**

To investigate the effect of nasal irrigation on the duration of symptoms and nucleic acid conversion in adults infected with the Omicron variant of COVID-19.

**Methods:**

This quasi-experimental study enrolled patients diagnosed with asymptomatic, mild, or moderate Omicron infection at the Shandong Public Health Clinical Center between April 1, 2022 and May 1, 2022. Patients were divided into two groups to receive Lianhua Qingwen granules and traditional Chinese medicine (TCM) prescriptions (conventional group) and 3% hypertonic saline nasal irrigation based on conventional treatment (nasal irrigation groups), respectively. Primary outcomes were symptom disappearance time and nucleic acid negative conversion time. Secondary outcomes were peripheral blood white blood cell (WBC), lymphocyte (LYM) count, neutrophil (NEU) count, C-reactive protein (CRP) level, and chest CT examination findings.

**Results:**

Eighty patients were included (40 patients/group). Multiple linear regression analysis showed that, after adjustment for comorbidities, smoking history, LYM count, and Ct values of N gene, the patients in the nasal irrigation group were more likely to get lower nucleic acid negative conversion time (β = −11.052, 95% CI: −8.277–13.827, *P* < 0.001) compared with the conventional group. The symptom disappearance time showed no significant improvement (*P* > 0.05). Subgroup analysis for treatment-naïve patients in the nasal irrigation group showed similar nucleic acid negative conversion time improvement (*P* = 0.038).

**Conclusion:**

Early nasal irrigation shortens the nucleic acid negative conversion time in adults infected with the Omicron variant but without improvements in symptom disappearance time.

## Key summary points

### Why carry out this study?

COVID-19 is a major public health burden in the world. Omicron variants are currently the most predominant strains. It can spread throughout communities and infect people who have been vaccinated or previously had COVID-19. It has been reported that nasal irrigation and oral rinse can reduce the risk of COVID-19 infection. Implementing effective treatment measures is vital in reducing its spread.

### What did the study ask?/What was the hypothesis of the study?

We hypothesized nasal irrigation could reduce the duration of symptoms and nucleic acid conversion in adults infected with the Omicron variant of COVID-19.

### What was learned from the study?

The nasal irrigation might shorten the nucleic acid negative conversion time in adults infected with the Omicron variant.

## Introduction

Coronavirus Disease 2019 (COVID-19) is an acute respiratory disease caused by SARS-CoV-2 (1, 2). The virus is transmitted person-to-person by symptomatic and asymptomatic persons through close contact *via* respiratory droplets (3). Clinically important features of SARS-CoV-2 pathogenesis include infection of cells *via* binding of the viral spike protein to angiotensin-converting enzyme 2 (ACE2) receptors, with cell entry requiring type 2 transmembrane serine protease to cleave ACE2 receptor and activate viral spike protein, infection nasal and bronchial epithelial cells and pneumocytes in early stage, and acceleration the viral replication and compromised epithelial-endothelial barrier integrity in later stages (1, 2, 4). COVID-19 was declared a global pandemic on March 11, 2020 (5). As of November 13, 2022, over 632 million cases, including over 6.5 million deaths, have been reported worldwide (6). Mortality secondary to COVID-19 is highly variable and related to age, the disease severity, and comorbidities: 0.3–2.3% for all patients; 10–23% for hospitalized patients; 26–50% for patients admitted to the ICU; 37–88% for patients requiring invasive mechanical ventilation or extracorporeal membrane oxygenation (ECMO) (1, 2).

The original Wuhan strain rapidly evolved into different variants, including Alpha (B.1.1.7 and Q lineages), Beta (B.1.351 and descendent lineages), Gamma (P.1 and descendent lineages), Delta (B.1.617.2 and AY lineages), Epsilon (B.1.427 and B.1.429), Eta (B.1.525), Iota (B.1.526), Kappa (B.1.617.1), Mu (B.1.621 and B.1.621.1), Zeta (P.2), 1.617.3, and Omicron (strain B.1.1.529) (7–9). The Omicron variant of 2019-nCoV was first detected in South Africa on November 9, 2021, and is currently the only variant of concern (7–10). The transmission capacity of the Omicron variant is 3–4 times that of the delta variant, a trait that allowed this variant to spread and predominate worldwide quickly (11). The Omicron variant was first detected in China on December 13, 2021, and caused sporadic outbreaks in many provinces and cities in 2022 (12).

At present, there are five versions of Omicron circulating, among which BA.2 is called the “invisible Omicron” due to the lack of “S gene shedding” (13). Compared with the original Omicron BA.1, BA.2 is the most resistant to the available vaccines and currently the most predominant variant (14). Since vaccines have been less effective against the omicron variant, new prevention methods and treatments are needed to combat its spread (15, 16).

The nasal passages are a significant site of COVID-19 colonization (17). Randomized controlled trials showed that nasal irrigation was effective against the common cold and other common upper respiratory infections (18, 19). Nasal irrigation with hypertonic saline could also improve the mucociliary clearance of COVID-19 (20). Nearly 90% of family doctors surveyed in one study reported prescribing nasal irrigation for one or more medical conditions (21).

While it is believed that the chloride ions in saline can allow cells to mount an antiviral defense by causing the production of hypochlorous acid (22), the therapeutic effect of nasal irrigation on Omicron infection has not been confirmed. Hence, this study aimed to investigate the effect of nasal irrigation on symptom duration and nucleic acid conversion in adults infected with the Omicron variant of SARS-CoV-2.

## Materials and methods

### Study design and participants

In this quasi-experimental study, patients diagnosed with asymptomatic, mild, or moderate Omicron infection at the Shandong Public Health Clinical Center from April 1 to May 1, 2022, were included during their quarantine observation period. The patients were diagnosed according to the “Diagnosis and Treatment Protocol for Novel Coronavirus Pneumonia (Trial Version 9)” by National Health Commission & State Administration of Traditional Chinese Medicine in China, and an expert panel arrived at the final diagnosis collaboratively. The patients were divided into four grades (asymptomatic, mild, moderate, and severe) according to the “Diagnosis and Treatment Protocol for Novel Coronavirus Pneumonia (Trial Version 9).” Asymptomatic patients have no clinical symptoms or signs. Mild cases are characterized by body aches, coughs, or mild fever and no apparent abnormalities in images. The moderate cases present mild pneumonia symptoms with radiological findings. The severe form of the disease can be characterized by severe pneumonia and hypoxia. This study did not include patients with severe COVID-19.

The virus strain was confirmed as the BA.2.2 variant strain by Jinan Center for Disease Control. Severe and critically ill patients, patients with abnormal heart, liver, and kidney function, nasal diseases not suitable for nasal irrigation, patients with coagulation disorders, severe upper respiratory tract infection, and acute middle ear infection, previously confirmed infections, and patients using anti-infection, anti-virus, or immunomodulatory therapies were excluded.

This study was reviewed and approved by the ethics committee of Shandong Public Health Clinical Center. All patients signed the informed consent form.

### Intervention

The patients were matched by age, sex, type of infection, time of infection, and 21 days Ct value of nasopharyngeal swab nucleic acid. Ct value is the number of cycles when the sample fluorescence exceeds a chosen threshold above the calculated background fluorescence; the lower the Ct value of a specific gene, the more the gene exists in the sample (23). The patients were divided into the conventional group (conventional treatment) and the nasal irrigation group (nasal irrigation treatment). Conventional treatment included Lianhua Qingwen granules and other traditional Chinese medicine (TCM) prescriptions. Traditional Chinese medicine (TCM) has exerted broad-spectrum effects on a series of influenza viruses by inhibiting viral propagation and regulating immune function (24). Several TCMs have been recommended by the Chinese National Health Commission to treat COVID-19, including Lianhua Qingwen granules (25–28). The Lianhua Qingwen granule components include Forsythia, honeysuckle, licorice, rhubarb, and other Chinese medicinal materials (27, 28). The nasal irrigation treatment included 3% hypertonic saline for nasal irrigation based on the conventional treatment. The Electric Children's SEAWATER NASAL CAVITY SPRAYER (Aide medical Co., Ltd., Guizhou, China) was used for nasal irrigation. The irrigation bottle was filled with 10 ml of hypertonic saline. The irrigation solution was applied to patients in a seated position. The nozzle was directed into one nasal cavity, the machine was turned on, and the irrigation was delivered for about 10 s. The patient was then told to blow out the rinse solution, and the process was repeated for the other nasal cavity. Irrigation was performed once every morning and before bed until the nucleic acid turned negative.

### Outcomes

The primary outcomes were symptom disappearance time and nucleic acid negative conversion time. Nasopharyngeal swab nucleic acid detection was performed using the Daan reagent (Daan Gene Co., Ltd, Guangzhou, China). From the seventh day of enrollment, the negative patients were retested on the second day (the interval must exceed 24 h), and two consecutive negatives were judged to be negative. The positive patients were tested at 1-day interval until they turned negative. Secondary outcomes were peripheral blood white blood cell (WBC) count, lymphocyte (LYM) count, neutrophil (NEU) count, C-reactive protein (CRP) level, and chest Computed Tomography (CT) results before and after treatment.

### Statistical analysis

SPSS 20.0 (IBM, Armonk, NY) was used for statistical analysis. Student's *t*-test and analysis of variance were used to analyze the quantitative data. The chi-square test was used to analyze the categorical data. Multiple linear regression analysis was performed to analyze the effect of nasal irrigation on nucleic acid negative conversion time. Subgroup analysis was also performed on treatment-naïve patients and the refractory patients (those who were isolated for observation for more than 20 days and whose Ct value of nasopharyngeal swab nucleic acid detection was <25) between the conventional group and nasal irrigation group. Two-sided *P* < 0.05 was considered statistically significant.

## Results

Eighty patients [mean age: 41.27 ± 7.85 years; 44 males (55%)] were included. Six patients had received two doses of 2019-nCoV vaccine (7.5%), and 62 patients had received three doses (77.5%). There were 40 patients in the conventional and nasal irrigation groups. The comorbidities (*P* = 0.033), smoking history (*P* = 0.016), LYM count (*P* = 0.034), and Ct values of the N gene (*P* < 0.001) were significantly different between the two groups (Table 1).

**Table 1:** Comparison of clinical characteristics between the routine group and nasal irrigation group.

**Variable**	**Routine group**	**Nasal irrigation group**	**P-value**
**Gender (** ***n*** **)**			**0.178**
Female	21	15	
Male	19	25	
Age	40.78 ± 13.78	41.10 ± 14.61	0.919
**Vaccination (** ***n*** **)**			
No	4	2	0.396
Yes	36	38	
**Basic disease (** ***n*** **)**			**0.033**
No	32	37	
Yes	8	3	
**Smoking history**			**0.016** ^*^
No	29	20	
Yes	11	20	
**Clinical symptoms (** ***n*** **)**			
Fever	13	12	0.809
Sore throat	11	5	0.094
Hoarseness	2	1	0.556
Dry cough	10	5	0.152
Expectoration	6	5	0.745
Physical decline	2	3	0.152
**Clinical typing (** ***n*** **)**			**0.251**
Asymptomatic	22	29	
Mild	7	5	
Moderate	11	6	
Lymphocyte count (^*^10^9^/L)	1.35 ± 0.79	1.18 ± 0.64	0.034^*^
**Ct value (nasopharyngeal swab)**			
N gene	17.27 ± 4.77	13.54 ± 4.12	< 0.001^**^
ORF gene	17.79 ± 3.63	16.67 ± 3.87	0.185

Data was expressed as number of cases or mean ± SD,

* *P* < 0.05,

** *P* < 0.01.

**Figure 1:**
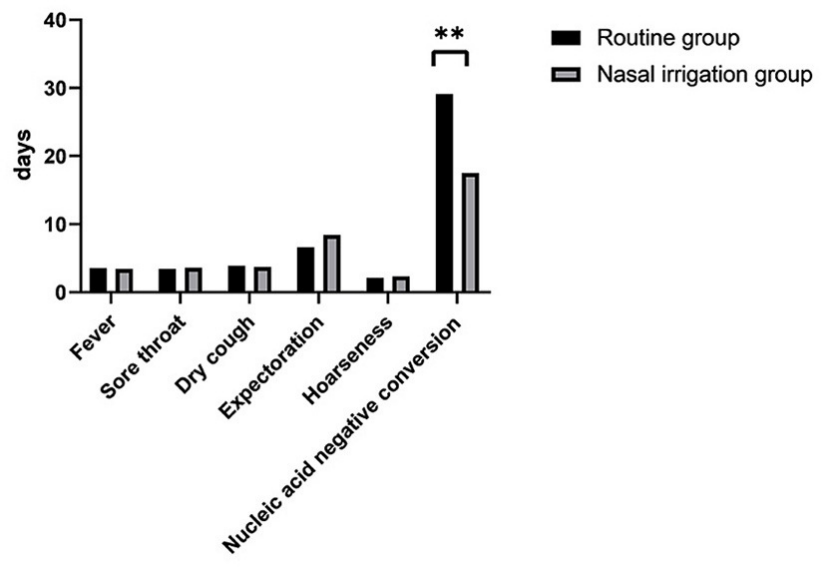
Time to the disappearance of symptoms and conversion of nucleic acids. Between the routine treatment and nasal irrigation groups. **P < 0.01.

The symptom disappearance time, including fever, sore throat, dry cough, expectoration, and hoarseness, between the two groups were comparable (all *P* > 0.05). Notably, the nucleic acid negative conversion time was significantly different (*P* < 0.001) between the conventional and nasal irrigation groups (Table 2, Figure 1). Multiple linear regression analysis showed that, after adjustment for comorbidities, smoking history, LYM count, and Ct values of N gene, the patients in the nasal irrigation group were more likely to have lower nucleic acid negative conversion time (β = −11.052, 95% CI: −8.277 to −13.827, *P* < 0.001) compared with the conventional group (Table 3). In addition, the changes in blood routine test and CRP indexes in the nasal irrigation and conventional groups were similar (all *P* > 0.05; Table 4).

**Table 2:** Primary outcomes: symptom disappearance time and nucleic acid negative conversion time (days).

**Outcomes (time, mean ±SD)**	**Routine group**	**Nasal irrigation group**	**P-value**
Fever	3.54 ± 1.39	3.44 ± 0.73	0.144
Sore throat	3.45 ± 1.29	3.60 ± 2.19	0.869
Dry cough	3.91 ± 2.70	3.71 ± 2.10	0.278
Expectoration	6.60 ± 3.21	8.40 ± 2.41	0.750
Hoarseness	2.12 ± 1.21	2.34 ± 1.71	0.324
Nucleic acid negative conversion	17.58 ± 7.31	29.10 ± 3.70	< 0.001^**^

Data was expressed as mean ± SD,

** *P* < 0.01.

**Table 3:** Multiple linear regression analysis for nucleic acid negative conversion time.

**Related factors**	**β value (95% CI)**	**P-value**
Routine treatment	Ref	–
Nasal irrigation treatment	−11.052(−8.277–−13.827)^a^	< 0.001^**^

a Adjusted for basic disease, smoking history, LYM count, Ct values of N gene.

** *P* < 0.01.

**Table 4:** Changes of blood routine test and CRP indexes in nasal irrigation group and routine group before and after treatment.

**Group**	**Time**	**WBC**	**LYN**	**NEU**	**CRP**
		(^*^10^9^/L)	(^*^10^9^/L)	(^*^10^9^/L)	**(mg/L)**
Nasal irrigation group	Before treatment	5.99 ± 1.46	1.15 ± 0.70	4.03 ± 1.26	23.27 ± 3.60
	After treatment	5.66 ± 1.38	1.38 ± 0.79	3.70 ± 1.04	22.81 ± 6.48
	*P*-value	0.076	0.046	0.114	0.684
Routine group	Before treatment	6.32 ± 1.84	1.37 ± 0.74	4.00 ± 1.62	23.56 ± 6.43
	After treatment	6.23 ± 1.54	1.41 ± 0.79	3.52 ± 1.35	23.10 ± 6.36
	*P*-value	0.638	0.691	0.026^*^	0.222

Data was expressed as mean ± SD, compared with before treatment,

* *P* < 0.05.

**Figure 2:**
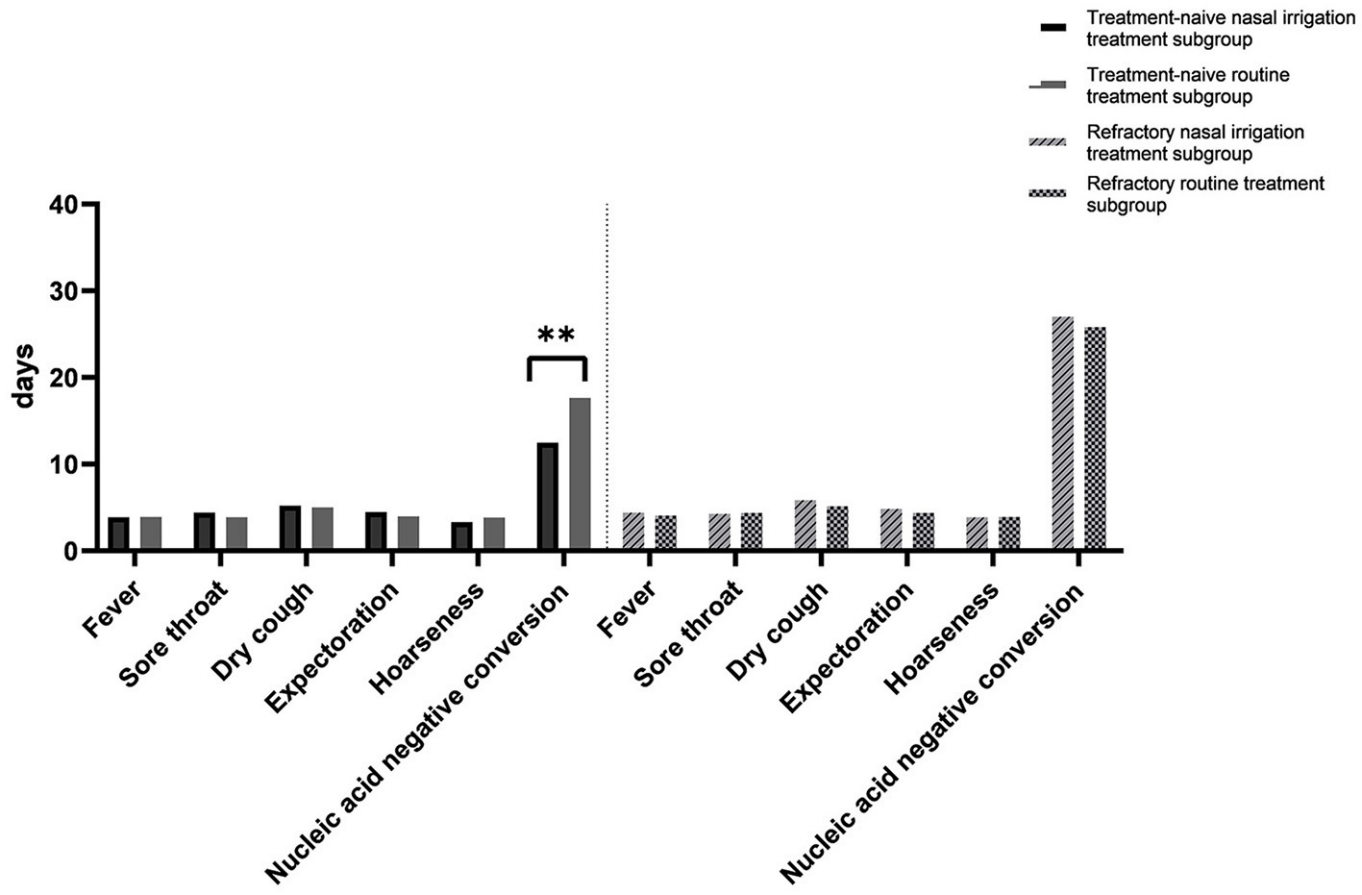
Time to the disappearance of symptoms and conversion of nucleic acids. Between the naïve and refractory subgroups. **P < 0.01.

In the subgroup analysis, the comparison of the clinical characteristics between the patients in the treatment-naïve and refractory subgroups were shown in Supplemental Table 1. Compared with the treatment-naïve patients in the conventional group, the average time of nucleic acid negative conversion of the treatment-naïve patients in the nasal irrigation group was significantly shortened (17.65 ± 2.18 vs. 12.48 ± 3.22 days, *P* < 0.001), while the average time of nucleic acid negative conversion of the refractory patients between the conventional and nasal irrigation groups was comparable (31.82 ± 4.75 vs. 32.02 ± 4.16 days, *P* = 0.888; Table 5, Figure 2).

**Table 5:** Primary outcomes: symptom disappearance time and time of nucleic acid negative conversion (days).

**Outcomes**	**Treatment-naive subgroup**			**Refractory subgroup**		
	**Nasal irrigation treatment**	**Routine treatment**	**P-value**	**Nasal irrigation treatment**	**Routine treatment**	**P-value**
Fever	3.87 ± 1.15	3.92 ± 1.09	0.889	4.32 ± 1.27	4.09 ± 1.18	0.556
Sore throat	4.24 ± 2.01	3.89 ± 1.15	0.503	4.28 ± 1.16	4.37 ± 1.25	0.815
Dry cough	5.22 ± 2.18	5.03 ± 2.27	0.789	5.86 ± 2.75	5.16 ± 2.33	0.391
Expectoration	4.84 ± 1.73	3.98 ± 1.16	0.073	4.85 ± 2.12	4.39 ± 2.03	0.488
Hoarseness	3.31 ± 1.01	3.86 ± 1.63	0.207	3.88 ± 1.29	3.92 ± 1.51	0.918
Nucleic acid negative	12.48 ± 3.22	17.65 ± 2.18	< 0.001^**^	27.02 ± 4.16	25.82 ± 4.75	0.888

Data was expressed as mean ± SD,

** *P* < 0.01.

Furthermore, multiple linear regression analysis showed that after adjustment for smoking history, clinical typing, Ct values of N gene, and LYM count, the treatment-naïve patients in the nasal irrigation group (β = −0.654, 95% CI: −0.997 to −0.512, *P* = 0.038) were more likely to get a lower time of nucleic acid negative conversion compared with those who in the conventional group, while the refractory patients in the nasal irrigation group showed no significant improvement (*P* = 0.324; Table 6).

**Table 6:** Multiple linear regression analysis for nucleic acid turning negative time in subgroup.

**Group**		**β value (95% CI)**	**P-value**
Treatment-naive subgroup	Routine treatment	Ref	–
	Nasal irrigation treatment	−0.654 (−0.997–−0.512)^a^	0.038^*^
Refractory subgroup	Routine treatment	Ref	–
	Nasal irrigation treatment	−0.178 (−0.341–0.113)^a^	0.324

a Adjusted for smoking history, clinical typing, Ct values of N gene, and LYM count,

* *P* < 0.05.

**Figure 3:**
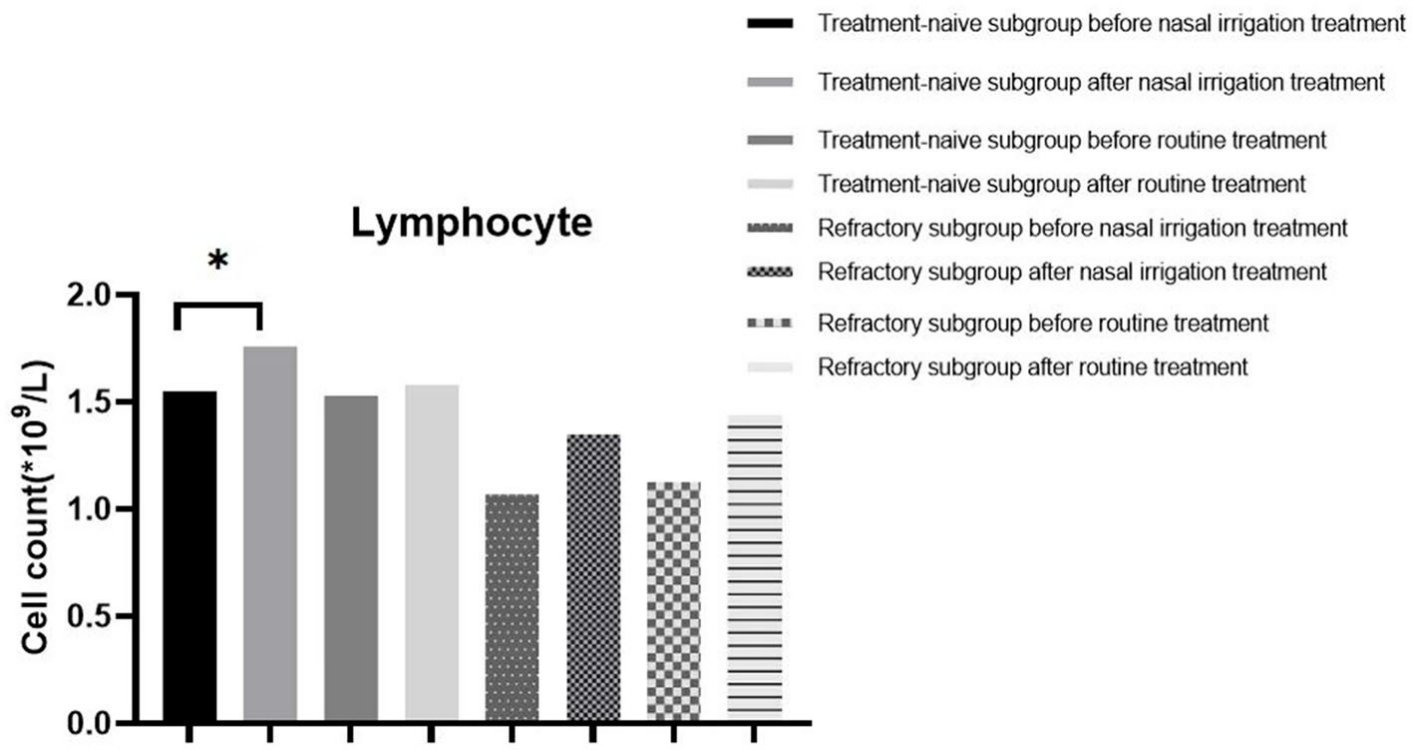
LYM count in the subgroup before and after different treatments. *P < 0.05.

**Figure 4:**
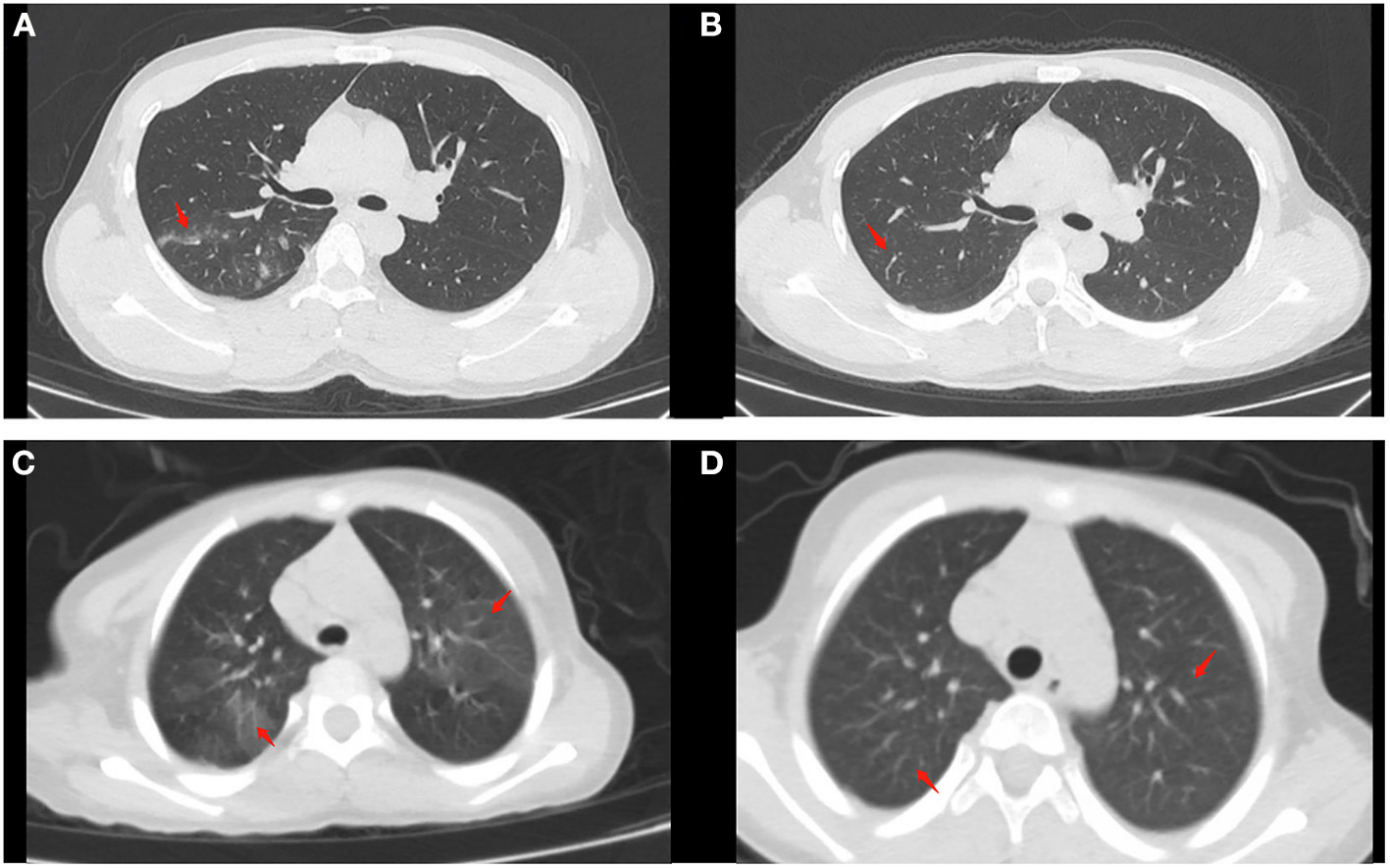
Chest CT findings of moderate cases before and after treatment (as arrow points). **(A)** Patchy high-density shadow can be seen in the posterior segment of the upper lobe of the right lung before treatment in naïve patients. **(B)** The high-density shadow subsided after treatment in naïve patients. **(C)** Patchy high-density shadow and blurred edges can be seen in the upper lobes of both lungs in a refractory patient. **(D)** No significant abnormalities can be seen after treatment in the refractory patient.

Additionally, the WBC count, NEU count, LYM count, and CRP level between the treatment-naïve and refractory groups before treatment were comparable (all *P* > 0.05). The LYM count and CRP level (*P* < 0.05) were significantly different between the treatment-naïve patients in the nasal irrigation group before and after treatment, while there were no statistical differences in the refractory patients before and after treatment (Table 7, Figure 3). The treatment-naïve and refractory patients showed significant improvement in inflammation, as shown by thoracic CT (Figures 4A–D).

**Table 7:** Changes of blood routine and CRP indexes in the treatment-naïve subgroup and refractory subgroup before and after treatment.

**Group**	**Time**		**WBC**	**LYN**	**NEU**	**CRP**
			(*10^9^/L)	(*10^9^/L)	(*10^9^/L)	**(mg/L)**
Treatment-naive subgroup	Nasal irrigation treatment	Before treatment	5.17 ± 0.28	1.55 ± 0.20	3.75 ± 0.32	25.62 ± 5.11
		After treatment	5.80 ± 0.36	1.76 ± 0.11^*^	3.26 ± 0.29	21.75 ± 4.28^**^
	Routine treatment	Before treatment	5.15 ± 0.28	1.53 ± 0.21	3.75 ± 0.33	27.25 ± 6.64
		After treatment	5.48 ± 0.27	1.58 ± 0.32	3.95 ± 0.41	27.86 ± 4.87
Refractory subgroup	Nasal irrigation treatment	Before treatment	5.83 ± 0.35	1.07 ± 0.13	3.97 ± 0.22	29.12 ± 5.28
		After treatment	5.52 ± 0.29	1.35 ± 0.16	3.76 ± 0.34	27.85 ± 5.12
	Routine treatment	Before treatment	5.68 ± 0.42	1.13 ± 0.18	3.85 ± 0.53	28.83 ± 4.25
		After treatment	5.26 ± 0.33	1.44 ± 0.31	3.86 ± 0.48	28.12 ± 4.46

Data was expressed as mean ± SD, compared with before treatment,

* *P* < 0.05,

** *P* < 0.01.

## Discussion

This study showed that patients in the nasal irrigation group were more likely to get lower nucleic acid negative conversion time than the conventional group, while the symptom disappearance time showed no significant improvement. Subgroup analysis for treatment-naïve patients in the nasal irrigation group showed similar nucleic acid negative conversion time improvement. This study supports that nasal irrigation might be a safe and effective treatment option for acute upper respiratory infections, including the highly infectious Omicron COVID-19 variant.

A total of 85% of the infected people in this study were fully vaccinated against SARS-CoV-2. After infection, the clinical manifestations of the Omicron mutant are usually mild, and there are few severe cases and deaths. The typical clinical symptoms were fever, dry cough, fatigue, and a small number of people had nasal congestion, runny nose, sore throat, vomiting, and diarrhea (29). In all, 60% of the patients in this study population were asymptomatic. In addition to fever and sore throat, the incidence of hoarseness was high, which was consistent with the reports of South Africa and other countries (30). This study showed that the average duration of symptoms in infected people was about 5.55 days.

The time to nucleic acid negative conversion in patients infected with Omicron varies from person to person. For Omicron, the average time is between 15 and 18 days, while for Delta, it is on average 25 days (31). This study showed that the time of nucleic acid-negative conversion of patients in the treatment-naïve subgroup without nasal irrigation was 17.65 ± 2.18 days, which is consistent with this report (31).

Studies confirmed that age over 60, smoking, poor basic health status, and decreased LYM count increase the risk of severe illness and death after Omicron infection (32). The host's innate and adaptive immune responses play important roles in defending against COVID-19 (33). Lymphocytopenia was reported in COVID-19 patients (34), and low LYM count (<0.95 × 10^9^/L) increased the mortality risk of patients with COVID-19 (35). This study showed that the LYM count was significantly different between the naïve and refractory subgroups. The patients in the refractory group had lower LYM count, and the LYM count improved when the COVID-19 nucleic acid turned negative. In asymptomatic and mild patients, smoking and low LYM count also increase the duration of Omicron infection, possibly because of poor immune function.

This study also showed that the level of the N gene in nasopharyngeal swabs was also related to the time to seronegative conversion. Encoded by the N gene, The N protein is one of the core components of the virus, and participates in immune regulation (36), which can bind to double-stranded RNA to resist RNA-mediated host antiviral responses (37). This might prolong the time of nucleic acid-negative conversion by inhibiting the body's antiviral response.

The COVID-19 virus primarily invades the upper respiratory tract in both symptomatic and asymptomatic patients, and the virus titer is significantly higher in the nose than that in the throat (38). Nasal irrigation with hypertonic saline has been reported to reduce nasal congestion, cough, and other symptoms caused by 2019-nCoV infection and shorten the infection period of novel coronavirus by an average of 2.5 days (39). This study showed that after nasal irrigation, the time of nucleic acid negative conversion was shortened by about 5 days on average in the treatment naïve group, but it did not affect the duration of symptoms. For patients who had an active viral infection for more than 3 weeks and the Ct value of nasopharyngeal swab nucleic acid was <25, the addition of nasal irrigation treatment failed to promote the negative conversion of the virus. It suggests that early nasal irrigation may shorten the time of seronegative conversion.

This study was limited primarily by its small size and single-center design. There were differences in the basic clinical characteristics among the groups of patients analyzed by this study despite randomization. It indicates that a larger random study is necessary to validate these results. In addition, refractory patients and treatment-naïve patients did not perform nasal irrigation at the same time, which may lead to a certain bias. Moreover, it might be prudent to test if saline nasal irrigation can impact the transmissibility of Omicron. Since it appears to reduce viral load when administered in the early stages of infection, it is reasonable to believe that it may help to prevent the spread of coronavirus.

## Conclusion

Early nasal irrigation shortens the nucleic acid negative conversion time in adults infected with the Omicron variant but without improvements in symptom disappearance time. The study may provide new insights into non-pharmacological intervention for the treatment of Omicron infection.

## Data availability statement

The original contributions presented in the study are included in the article/Supplementary material, further inquiries can be directed to the corresponding authors.

## Ethics statement

The studies involving human participants were reviewed and approved by Shandong Public Health Clinical Center. The patients/participants provided their written informed consent to participate in this study.

## Author contributions

LL carried out the studies, participated in collecting data, and drafted the manuscript. LS and CZ performed the statistical analysis and participated in its design. SX and CL participated in the acquisition, analysis, or interpretation of the data. All authors read and approved the final manuscript.
