# Supplementary material for: Effect of nasal irrigation in adults infected with Omicron variant of COVID-19: A quasi-experimental study
Source: Front Public Health. 2023 Jan 9;10:1046112. doi: 10.3389/fpubh.2022.1046112 (PMC9868717; doi:10.3389/fpubh.2022.1046112)
Supplement: Supplementary file 1 [file Table_1.docx]

Supplemental Table 1 Comparison of clinical characteristics between treatment-naive subgroup and refractory subgroup.

| Variable | Treatment-naive subgroup | | Refractory subgroup | | P value |
| --- | --- | --- | --- | --- | --- |
|  | Routine treatment | Nasal irrigation treatment | Routine treatment | Nasal irrigation treatment |  |
| Gender (n) |  |  |  |  | 0.091 |
| Female | 8 | 13 | 10 | 5 |  |
| Male | 12 | 7 | 10 | 15 |  |
| Age | 41.55±12.30 | 40.00±15.41 | 41.15±12.39 | 41.05±16.87 | 0.988 |
| Vaccination (n) |  |  |  |  | 0.499 |
| No | 3 | 1 | 0 | 2 |  |
| Yes | 17 | 19 | 20 | 18 |  |
| Basic disease (n) |  |  |  |  | 0.037* |
| No | 20 | 15 | 18 | 14 |  |
| Yes | 0 | 5 | 2 | 6 |  |
| Smoking history |  |  |  |  | 0.004** |
| No | 15 | 13 | 16 | 6 |  |
| Yes | 5 | 7 | 4 | 14 |  |
| Clinical symptoms (n) |  |  |  |  |  |
| Fever | 5 | 8 | 5 | 7 | 0.734 |
| Sore throat | 7 | 4 | 2 | 3 | 0.297 |
| Hoarseness | 0 | 2 | 0 | 1 | 0.611 |
| Dry cough | 3 | 7 | 2 | 3 | 0.23 |
| Expectoration | 0 | 6 | 3 | 2 | 0.05 |
| Physical decline | 0 | 2 | 0 | 0 | 0.241 |
| Clinical typing (n) |  |  |  |  | 0.551 |
| Asymptomatic | 10 | 12 | 16 | 13 |  |
| Mild | 3 | 4 | 2 | 3 |  |
| Moderate | 7 | 4 | 2 | 4 |  |
| Lymphocyte count | 1.27±0.74 | 1.44±0.86 | 1.04±0.65 | 1.31±0.62 | 0.385 |
| Ct value (nasopharyngeal swab) |  |  |  |  |  |
| N gene | 17.55±4.47 | 17.00±5.14 | 13.20±4.78 | 13.87±3.43 | 0.005** |
| ORF gene | 18.88±3.14 | 16.70±3.82 | 16.69±3.82 | 16.65±4.01 | 0.162 |

Note: data was expressed in the form of number of cases (n) or mean ± SD,*P<0.05, **P<0.01.
